# Observational studies provide insufficient data for a reliable meta-analysis: a call to revise the current guidelines

**DOI:** 10.1186/s13643-023-02435-7

**Published:** 2024-01-02

**Authors:** Amirmohammad Toloui, Mahmoud Yousefifard

**Affiliations:** Physiology Research Center, Iran University of Medical Sciences, Tehran, Iran

Dear Editor,

Systematic reviews and meta-analyses provide the highest level of evidence in the scientific literature through a comprehensive overview of the existing evidence [1, 2]. Systematic reviews and meta-analyses were originally developed to pool data from randomized controlled trials (RCTs) [3]; however, they have since expanded to incorporate observational studies, leading to their inclusion in the evidence-based pyramid. A preliminary search in the Medline database (May 2023), exploiting filters for systematic reviews, meta-analyses, and relevant keywords for observational articles, yields about 35,000 articles. This substantial number reflects the increasing utilization of systematic reviews and meta-analyses for pooling the results of observational studies.

Unlike RCTs, the case/control or expose/non-exposed groups in most observational studies differ in various underlying variables. To explore the independent association between the exposure and a binary outcome (such as death, unfavorable outcomes, or the need for hospitalization), observational studies report the adjusted results by employing multivariate models, such as stepwise multiple logistic regressions, general linear models, and Cox hazard regressions. These models report the adjusted statistics in odds ratio (OR), relative risk (RR), or hazard ratio (HR). The approach that observational studies currently undertake to report these findings introduces significant bias in the meta-analyses of observational studies.

In automated model selection methods (such as stepwise, backward, and forward models) the statistical software includes or excludes variables based on their significance level. While the SPSS statistical program provides a separate table for the models’ excluded variables, STATA does not report an excluded table by default. In light of that, most observational studies only report the significant variables in their multivariable analyses, neglecting the output of non-significant results. This selective reporting introduces serious bias in attempts to pool data. It is noteworthy that even though controversies exist in terms of the robustness and a possible bias in the results of stepwise models, they are still widely used in the scientific literature as the most commonly utilized automated model selection methods [4–7].

We hereby use a hypothetical scenario to better illustrate. Consider that a meta-analysis aims to examine the relationship between fast food consumption and cancer by pooling the data from observational studies. Among the 20 included studies, 12 find a significant relationship between fast food consumption and cancer occurrence in a stepwise multivariate logistic regression model and report the adjusted ORs. In the remaining 8 articles, fast food consumption shows no independent relationship with cancer and is consequently excluded from the multivariable model with no reported adjusted ORs. If the researcher decides to conduct a meta-analysis based on the 12 studies with the adjusted ORs, the pooled effect size is undoubtedly overestimated. On the other hand, if the researcher decides to perform the meta-analysis on the unadjusted results (univariate analyses), the independence of the relationship between fast food consumption and cancer occurrence will remain unclear. Conclusively, neither of the provided pieces of evidence is accurate nor reliable. It is important to note that the unadjusted OR cannot be pooled with the adjusted OR, since the estimated effect size of a single variable in a univariate analysis is considerably different than its reported results in the multivariate analysis.

Another common issue in observational studies is the inconsistency in reporting the final summary results in multivariate regressions. As mentioned earlier, observational studies primarily use OR, RR, or HR to report the outputs of multivariate models. In certain circumstances, OR and RR can be pooled in the meta-analysis: if the OR is close to 1 and the prevalence of the outcome is below 10% [8]. Nevertheless, to the extent of our knowledge, there is no standard method to substitute or transform HR into OR/RR. The disparity in the reported statistics leads to the inevitable exclusion of some of the included studies from a meta-analysis.

In order to address these sources of bias, researchers conducting original observational studies should report the effect sizes and the 95% confidence intervals of non-significant variables in their multivariate analyses (for example, in a separate table titled “non-significant variables of the multivariate analysis”) either in the main text or in the supplementary materials of their articles. Also, observational studies should report OR, RR, and HR (if the time-to-event data is available), enabling the pooling of data from all included articles.

To illustrate this more, Strengthening the Reporting of Observational Studies in Epidemiology (STROBE) could be modified by adding a section (d) to the “main results” stating “Provision of the significant and non-significant variables of multivariate models” [9]. Also, “Provision of different estimates of effect sizes, including OR, RR, and HR (if applicable)” could be added to the “other analyses” section. The same recommendation applies to the Standards for the Reporting of Diagnostic Accuracy Studies (STARD), adding “Provision of the significant and non-significant variables of multivariate models” to the “test results” section. Also, in order to provide different estimates of effect sizes, the “analysis” section of “methods” along with the “test results” could be adjusted by adding the statement “Provision of different estimates of effect sizes, including OR, RR, and HR (if applicable)” [10].

Even though our focus was to rectify this issue at the level of the original observational studies, the evaluation of this selective reporting should be thoroughly implemented as a domain into the guidelines for the risk of bias assessment of observational studies (such as NHLBI or Newcastle–Ottawa Scale). For example, “Were non-significant variables of multivariate models reported?” could be added to the NHLBI tools, and “Provision of the significant and non-significant variables of multivariate models” could be added to the “comparability” sections of the Newcastle–Ottawa Scale.

Lastly, since the initial step in a systematic review/meta-analysis is the screening process by the title/abstract of the acquired records from the systematic search, authors of the original articles should at least report the names of all variables in their abstract—regardless of the significance level in the final multivariate analysis—to avoid missing any potentially relevant article in a systematic review/meta-analysis on observational studies that report exposure-outcome or prognostic relations. As a recommendation, “Indicate all included variables of the analysis, with either names or their categories” could be added to the “Abstract” section in both STROBE and STARD.

## Highlights

Systematic reviews and meta-analyses provide the highest level of evidence in health sciences. With the advancement of this research methodology to pool data obtained from observational studies, a comprehensive approach toward the best statistical method of reporting the findings is essential. We hereby point out a significant cause for bias in meta-analyses of observational studies due to the insufficient reports of findings. Not only authors of observational studies should report the significant variables from multivariate analyses, but a robust meta-analysis requires reports from the non-significant variables too. In addition, different means of reporting the estimated effect sizes of multivariable analyses prohibit a thorough data pooling strategy. We recommend modifications to the guidelines of how to conduct observational studies to address these issues.
